# Associations of childhood overweight and obesity with upper-extremity fracture characteristics

**DOI:** 10.1097/MD.0000000000025302

**Published:** 2021-05-07

**Authors:** Derek T. Nhan, Arabella I. Leet, R. Jay Lee

**Affiliations:** a Shriners Hospital for Children Honolulu, Honolulu, HI; b The Johns Hopkins University, Baltimore, MD; c Department of Orthopedic Surgery, University of Washington, Seattle, WA.

**Keywords:** children, fracture, obesity, physeal involvement, upper extremity

## Abstract

Childhood obesity is a growing epidemic in the United States, and is associated with an increased risk of lower-extremity physeal fractures, and fractures requiring operative intervention. However, no study has assessed the risk upper extremity physeal fractures among overweight children. Our purpose was to compare the following upper-extremity fracture characteristics in overweight and obese children with those of normal-weight/underweight children (herein, “normal weight”): mechanism of injury, anatomical location, fracture pattern, physeal involvement, and treatment types. We hypothesized that overweight and obese children would be higher risk for physeal and complete fractures with low-energy mechanisms and would therefore more frequently require operative intervention compared with normal-weight children.

We performed a cross-sectional review of our database of 608 patients aged 2 to 16 years, and included patients who sustained isolated upper-extremity fractures at our level-1 pediatric tertiary care center from January 2014 to August 2017. Excluded were patients who sustained pathologic fractures and those without basic demographic or radiologic information. Using body mass index percentile for age and sex, we categorized patients as obese (≥95th percentile), overweight (85th to <95th percentile), normal weight (5th to <85th percentile), or underweight (<5th percentile). The obese and overweight groups were analyzed both separately and as a combined overweight/obese group. Demographic data included age, sex, height, and weight. Fractures were classified based on fracture location, fracture pattern (transverse, comminuted, buckle, greenstick, avulsion, or oblique), physeal involvement, and treatment type. Of the 608 patients, 58% were normal weight, 23% were overweight, and 19% were obese. There were no differences in the mean ages or sex distributions among the 3 groups.

Among patients with low-energy mechanisms of injury, overweight/obese patients had significantly greater proportions of complete fractures compared with normal-weight children (complete: 65% vs 55%, *P* = .001; transverse: 43% vs 27%, *P* = .006). In addition, the overweight/obese group sustained significantly more upper-extremity physeal fractures (37%) than did the normal-weight group (23%) (*P* = .007).

Compared with those in normal-weight children, upper-extremity fracture patterns differ in overweight and obese children, who have higher risk of physeal injuries and complete fractures caused by low-energy mechanisms.

Level of Evidence: Level III, retrospective comparative study.

## 1 Introduction

Obesity is a major epidemic in the United States and is occurring at younger ages than in the past.^[1]^ Estimates show a 4-fold increase in the prevalence of obesity since the 1970s. Moreover, recent data indicate that >30% of US children and adolescents are overweight,^[2]^ and of those, 16% are obese. Obesity rates are growing fastest among boys aged 6 to 19 years.^[3]^ Predictive models suggest nearly 30% of children will be obese by 2030.^[3]^

Among children, upper-extremity fractures represent more than half of all bony injuries.^[4]^ Studies have examined the association of pediatric obesity with elbow, forearm, and femur fractures and have found that obese children are at greater risk of these injuries compared with nonobese children. Studies have found that, compared with nonobese children, obese children sustain more severe fractures and that these fractures are more likely to require complex surgical intervention.^[5–9]^

Large pediatric trauma registry data may not be generalizable to all types of extremity fractures, especially those from lower-energy mechanisms (eg, sports injuries, ground-level falls, and playground injuries) that represent a large proportion of fractures in children. To our knowledge, no study has evaluated the risk of physeal involvement, stratified by body mass index (BMI), for upper-extremity fractures in children.

The purpose of our study was to evaluate the associations of excess weight with upper-extremity fracture patterns by comparing overweight and obese children with normal-weight children treated at an urban pediatric tertiary care center in terms of mechanism of injury, anatomical location, fracture pattern, physeal involvement, and treatment types. We hypothesized that overweight and obese children would be at higher risk for physeal fractures, and that these would tend to occur at lower-energy mechanisms compared with their normal-weight counterparts. We hypothesized that children with excess weight will have distinct upper-extremity fracture patterns compared to normal-weight children.

## 2 Materials and methods

Institutional review board approval was obtained to develop a database and review all patients with fractures evaluated in the emergency department or clinic of a level-1 pediatric tertiary care center from January 2014 to August 2017. Using these data, we identified records of patients aged 2 to 16 years when they were treated for upper-extremity fracture. Data collected from the time of injury included patient age; sex; height and weight recorded within 1 month of the injury; mechanism of injury; bone and anatomic location involved (proximal humerus, humeral diaphysis, distal humerus, radius, ulna, both bones of forearm, or hand); fracture pattern (avulsion, buckle, comminuted, greenstick, oblique, or transverse); physeal involvement; and treatment type. Fracture patterns were further classified as complete (transverse, comminuted, or oblique) or incomplete (buckle, avulsion, or greenstick). Mechanisms of injury were graded as high-energy or low-energy. High-energy mechanisms included motor vehicle accidents, gunshot wounds, and falls from a height >4 feet. All other mechanisms, such as playground injuries, sports injuries, or ground-level falls, were considered low-energy. Treatment type was classified as surgical or nonsurgical.

**Figure 1:**
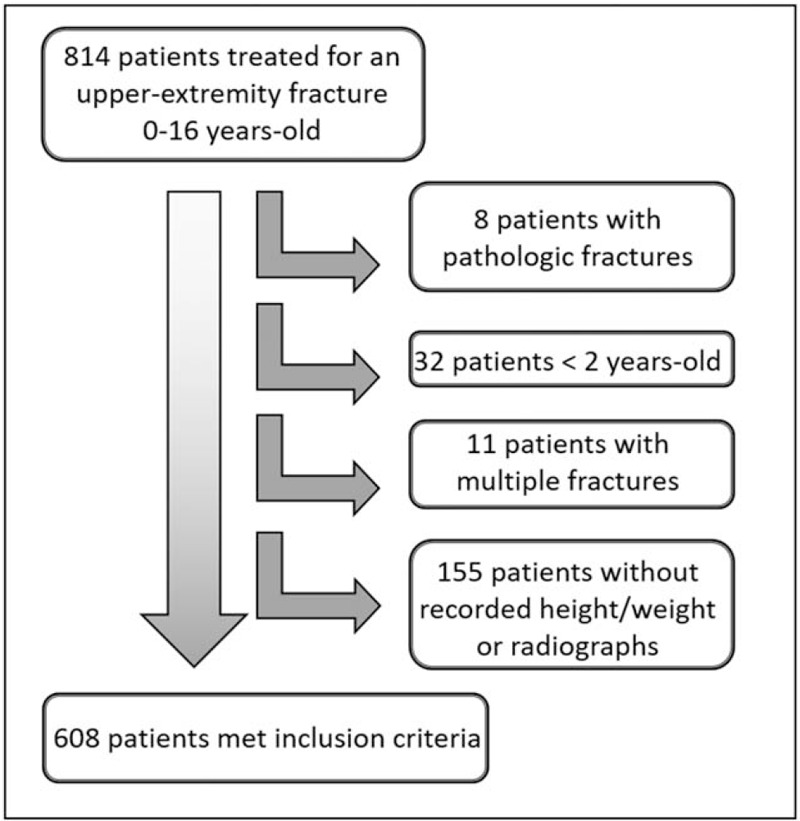
Patient selection flowchart. After exclusions, 608 of 814 patients (75%) met our inclusion criteria.

During the study period, 814 children were treated for a single upper-extremity fracture, of whom 608 met our inclusion criteria (Fig. 1). We excluded children with pathologic fractures, such as fractures occurring through a bony lesion or those in children with known neuromuscular disorders. We also excluded patients without recorded height or weight within the specified period and those without initial radiographs obtained at our institution. Patients younger than 2 years were excluded because they are not included in the Centers for Disease Control and Prevention's BMI-for-age charts. Patients with multiple fractures were also excluded.

Of the patients included in the study, 23% were overweight and 19% were obese. The other 58% were classified as normal weight or underweight. The mean (± standard deviation) age of all patients was 8.9 ± 3.5 years, and 63% were male. There were no significant differences in mean age (*P* = .422), sex distribution (*P* = .454), or race/ethnicity (*P* = .142) between BMI groups (Table 1).

**Table 1:** Patient demographic characteristics by body mass index percentile-for-age and sex.

	N (%)^∗^			
Characteristic	Normal-weight/underweight (n = 354)	Overweight (n = 137)	Obese (n = 117)	*P*
Age, y	8.8 ± 3.6^†^	9.3 ± 3.4^†^	9.0 ± 3.1^†^	.422
Sex				
Male	223 (63)	90 (66)	68 (58)	.454
Female	131 (37)	47 (34)	49 (42)	
Race/ethnicity				
African American	134 (38)	50 (36)	50 (43)	
Asian	22 (6.2)	3 (2.2)	3 (2.6)	.142
White	168 (47)	59 (43)	43 (37)	
Hispanic	2 (0.56)	3 (2.2)	3 (2.6)	
Other	10 (2.8)	14 (10)	7 (6.0)	
Not specified	18 (5.1)	8 (5.8)	11 (9.4)	

∗ Percentages within categories may not sum to 100% because of rounding error.

† Data presented as mean ± standard deviation.

### 2.1 Statistical analysis

Using BMI percentile for age and sex, we categorized patients as obese (≥95th percentile), overweight (85th to <95th percentile), normal weight (5th to <85th percentile), or underweight (<5th percentile). Because of the small number of underweight patients, they were grouped with those of normal weight (herein, “normal-weight” group). We analyzed overweight and obese patients both separately and as a combined overweight/obese group and compared them with normal-weight patients. Subgroup analyses were performed by comparing each BMI group across the variables measured and considering physeal fractures the primary outcome of interest. Demographic and fracture characteristics were compared between groups using analysis of variance with post-hoc Tukey honest significant difference tests for continuous data and Pearson *χ*^2^ tests for categorical data, with an alpha-level of 0.05.

## 3 Results

### 3.1 Mechanisms of injury

Ninety-six percent of all upper-extremity fractures in our cohort were caused by a low-energy mechanism. Low-energy mechanisms were the underlying cause for at least 95% of all upper-extremity fractures, irrespective of BMI subgroup. No difference was observed in the distribution of upper-extremity fractures secondary to low-energy versus high-energy mechanisms when comparing across the BMI classes (*P* = .500) (Table 2).

**Table 2:** Fracture characteristics by body mass index percentile-for-age and sex.

	N (%)^†^			
Parameter	Normal-weight/underweight (n = 354)	Overweight (n = 137)	Obese (n = 117)	*P*
Mechanism of injury				
Low-energy	336 (95)	133 (97)	113 (97)	.500
High-energy	18 (5.1)	4 (2.9)	4 (3.4)	
Physis involved				
Yes and low-energy mechanism	77 (23)	42 (32)	42 (37)	.007
Yes and high-energy mechanism	4 (22)	1 (25)	1 (25)	
Treatment				
Operative	64 (18)	22 (16)	18 (15)	.514
Nonoperative	290 (82)	115 (84)	99 (85)	
Bony location				
Diaphysis humerus	16 (4.5)	4 (2.9)	5 (4.3)	.068
Distal humerus	138 (39)	38 (28)	43 (37)	
Forearm (radius, ulna, both)	177 (50)	82 (60)	61 (52)	
Hand	21 (5.9)	11 (8.0)	7 (6.0)	
Proximal humerus	2 (0.56)	2 (1.5)	1 (0.85)	
Fracture pattern				
Avulsion	5 (1.4)	2 (1.5)	1 (0.85)	.040
Buckle	109 (31)	47 (34)	32 (27)	
Comminuted	67 (19)	22 (16)	23 (20)	
Greenstick	40 (11)	12 (8.8)	10 (8.5)	
Oblique	40 (11)	17 (12)	10 (8.5)	
Open	9 (2.5)	2 (1.5)	1 (0.85)	
Transverse	95 (27)	47 (34)	50 (43)	

^∗^Percentages calculated as a fraction of described variable among fractures for that BMI class.

† Percentages within categories may not sum to 100% because of rounding errors.

### 3.2 Anatomical locations

Forearm fractures (involving radius, ulna, or both bones of the forearm) accounted for the largest proportion of fractures, irrespective of BMI group (53%), followed by distal humerus fractures (36%). This order of frequency was consistent within each BMI group. There were no differences in the frequency of fractures by anatomic location between the BMI groups (*P* = .068).

### 3.3 Fracture patterns

**Figure 2:**
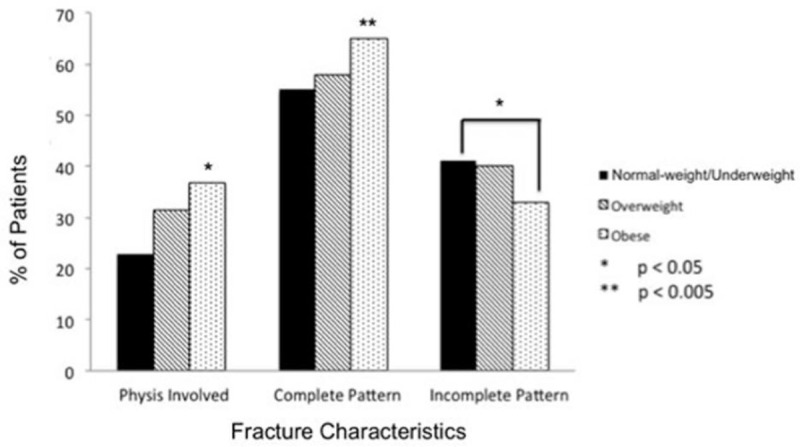
Differences in upper-extremity fracture characteristics by body mass index group in 608 children. The obese group had a significantly greater proportion of physeal injuries compared with the normal-weight/underweight and overweight groups. Obese children also had a greater proportion of complete fractures, whereas normal-weight/underweight children had a greater proportion of incomplete fractures.

Complete fractures represented 65% of fractures in obese patients, which was a significantly greater proportion than in overweight patients (58%) and normal-weight patients (55%) (both, *P* = .001). Conversely, normal-weight children sustained a greater proportion of incomplete fractures (41%) compared with obese patients (33%) (*P* = .006; Fig. 2).

Transverse fractures were the most common type in the obese group (43%), which was a significantly higher proportion than those in the normal-weight (27%) and overweight (34%) groups (both, *P* = .001). Obese children had 1.5 times higher risk of transverse fractures than did normal-weight children (relative risk = 1.5, 95% confidence interval: 1.2–2.0). Conversely, buckle fractures were the most frequent pattern in the normal-weight group (30%) and second most common in the overweight group (32%), although these differences were not significant compared with the obese group (25%).

### 3.4 Physeal involvement

Obese patients had a higher proportion of physeal injury (37%) compared with any other BMI group (*P* = .010). In addition, obese patients had significantly more physeal involvement from low-energy mechanisms (37%) than did normal-weight patients (23%) (*P* = .003), which represented >1.6 times greater relative risk (95% confidence interval: 1.2–2.3). This difference was also significant when comparing the obese/overweight group (34%) with the normal-weight group (*P* = .003) (Fig. 2) No differences were observed with respect to physeal injury risk from high-energy mechanisms among the BMI subgroups (25% in obese group, 22% in overweight group, and 22% in normal/underweight group; *P* = .988).

### 3.5 Treatment types

There were no differences in frequency of surgical intervention across BMI groups (15% for obese, 16% for overweight, and 18% for normal weight) (*P* = .514).

## 4 Discussion

Obese/overweight patients sustained more physeal fractures, and these fractures occurred more often from low-energy mechanisms and in a complete, transverse pattern compared with normal-weight patients. This finding has clinical implications in providing awareness regarding the types of fractures to be concerned for in overweight children, and to subsequently develop treatment prevention strategies targeted at preventing these fractures in this patient subgroup.

Our observation that obese children have a greater propensity for physeal involvement in upper-extremity fractures secondary to low-energy mechanisms compared with normal-weight and underweight children adds to the known fracture risk related to obesity.^[10,11]^ Possible reasons for this include relative bone mineral content and tendency for decreased activity in overweight and obese children. Studies have shown normal to increased bone mineral content in overweight and obese children; despite this, obese children may have relatively less bone mass for their bone size and body weight, and their increased bone density may be insufficient to overcome the substantially greater force when they fall.^[12,13]^ In addition, although we are unable to make statements about behavioral patterns in obese children, a dose-dependent association has been suggested between proxies for sedentary lifestyle behaviors (eg, “screen time”) and childhood obesity and fracture risk. Valerio et al^[14]^ suggested that inactivity may predispose to poorer postural balance, decreased coordination, and thus increased risk of falling. Further research is necessary to explore the associations of obesity with the risk of falling and other low-energy mechanisms of injury

When obese children sustained upper-extremity fractures, they were significantly more likely to sustain complete fractures compared with the incomplete patterns more frequently observed in normal-weight/underweight patients. It is well established that excess weight, in addition to metabolic changes, causes increased mechanical stress on bone,^[15]^ as well as a remodeling effect, whereby the bone thickens in response to this stress. In light of previous findings that show that obese children fall with greater force than do nonobese children from equal heights, this supports the reasoning^[12]^ that obese children are more likely to have through-and-through bony injuries rather than the buckle or greenstick patterns we observed in normal-weight/underweight children. It is likely that the increased stress and force from low-energy mechanisms outweighs any compensatory increase in bone mineral content. Other possible explanations for the higher risk of complete, transverse fractures in obese children include reduced dietary calcium intake compared with normal-weight children and hormonal effects,^[16]^ such as the known increased levels of free sex hormones,^[17]^ which reduce the bicortical strength of bone.

Research addressing the association of obesity with physeal injuries is sparse. Using a multicenter trauma registry, Gilbert et al^[18]^ reported that obese children were more likely to sustain physeal fractures of the femur or tibia compared with nonobese patients. Physeal injury is concerning because of its potential to cause angular deformities and limb-length discrepancies, which may require further orthopedic intervention.^[19–21]^ The combination of the higher incidence of preceding low-energy events and greater physeal involvement in upper-extremity fractures in obese children compared with all other children suggests a biomechanical component. Given that the physis is the weakest location within a bone, greater deceleration loads seen in obese children from a fall, sport, or playground injury would cause them to experience a greater force, resulting in failure at this weakest section. In a matched cohort study, Skaggs et al^[22]^ found that overweight children had a smaller cross-sectional area of the distal radius than did normal-weight children, suggesting that the greater force of a fall would be distributed across a smaller area in overweight children. According to biomechanical studies of load, even transient, abrupt increases in compressive forces can cause physeal thinning, particularly at sites of proliferation and hypertrophy.^[23–26]^ This could explain the increased physeal fracture frequency in both the upper extremities in the obese group compared with the other BMI groups.

Our study is limited by analysis of a single institution's patients. Although we report a slightly higher rate of obesity (19%) compared with the national average (16%), our rate aligns with data from schoolaged children in our region, who have an obesity rate of approximately 22% and an obese/overweight rate of 40%.^[27]^ Compared with other studies relying on trauma registries, our comprehensive database allowed us consistently to include height, weight, and mechanism of injury data while capturing fractures that present to low-acuity outpatient clinics. Because we found that obese children have a high frequency of physeal injuries from low-energy mechanisms, level-1 trauma registry data may not capture the true association between obesity and fracture risk. Therefore, our results may overestimate the national risk of physeal injury. In addition as our study was not population-based and thus unable to estimate incidence and odds ratios, our study would benefit from a larger sample size and multicenter nature to achieve the necessary statistical power to analyze subclasses of physeal fracture and treatment outcomes, especially to discern differences between BMI subgroups.

## Author contributions

**Conceptualization:** Arabella I. Leet, R. Jay Lee.

**Data curation:** Derek Tang Nhan, R. Jay Lee.

**Formal analysis:** Derek Tang Nhan.

**Funding acquisition:** Arabella I. Leet.

**Investigation:** Derek Tang Nhan, Arabella I. Leet.

**Methodology:** Derek Tang Nhan.

**Project administration:** R. Jay Lee.

**Validation:** Derek Tang Nhan, R. Jay Lee.

**Visualization:** Derek Tang Nhan.

**Writing – original draft:** Derek Tang Nhan.

**Writing – review & editing:** R. Jay Lee.
